# DAPA-HF trial signals the birth of ‘diabetic cardiology’ and more

**DOI:** 10.21542/gcsp.2020.22

**Published:** 2020-11-30

**Authors:** Kerolos Wagdy, Peter Selwanos

**Affiliations:** 1 Adult cardiology department, Aswan Heart Centre, Aswan, Egypt

## Introduction

**Figure 1.**
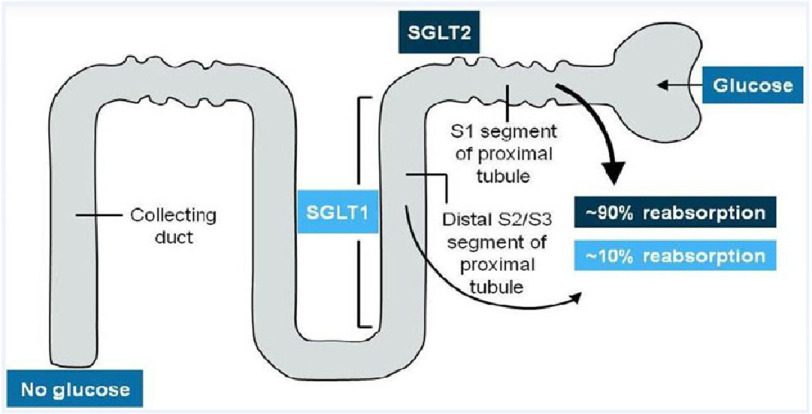
The mechanism of action of SGLT2 inhibitors.

Sodium-glucose co-transporter-2 (SGLT2) inhibitors are relatively new class of antihyperglycemic medication that is well established in the management of type 2 diabetes mellitus (DM)^1^. It has a unique mechanism of action that targets the kidneys through inhibiting 90% of glucose reabsorption Figure 1.

In addition to its effect on glycemic control, its action is associated with natriuresis and diuresis, weight reduction, blood pressure reduction, reduction of diabetes-related ventricular remodeling, and potential cardiovascular benefits^2^. The three agents of the SGLT2 inhibitors, which have been approved from FDA, are empagliflozin, canagliflozin and dapagliflozin.

The most common side effects of SGLT2 inhibitors are polyurea, volume depletion (due to their osmotic effect) and genitourinary infections (as they can cause high glucose in the urine). Serious infections are rare, although known to include urosepsis and pyelonephritis. Recently, however, the FDA has warned about cases of Fournier’s gangrene associated to SGLT2 inhibitor usage^3^.

Other rare side effects are euoglycemic diabetic ketoacidosis and hypoglycemia, particularly when SGLT2 inhibitors are concurrently prescribed with insulin or sulphonylureas.

Over the last five years, SGLT2 inhibitors have had an exciting and growing role in cardiovascular (CV) protection as reported in three landmark trials (EMPA-REG, CANAVAS and DECLARE-TIMI 58 trials). For the first time in the history of type-2 diabetes, we have data which indicates CV benefits from the use of glucose-lowering drugs in patients with CV disease or at CV risk. Trial evidence strongly suggests that these new drugs should be recommended for patients with type-2 diabetes mellitus at risk of cardiovascular disease, or with co-morbid cardiovascular disease^4^.

In the CANVAS clinical trial, canagliflozin reduced the risk of adverse major cardiovascular events (CV death, non-fatal myocardial infarction, and stroke), hospitalization for heart failure, and renal events in type-2 diabetic patients with a history - or at risk - of cardiovascular events^5,6^.

The EMPA-REG OUTCOME trial showed that empagliflozin reduced mortality, hospitalization for heart failure, and renal events in type-2 diabetic patients with cardiovascular risk.^7^

The DECLARE-TIMI 58 trial of dapagliflozin included patients who were at risk but didn’t have established cardiovascular disease, and reported a significant reduction in risk for hospitalization for heart failure and risk for renal composite; however it didn’t reduce the risk for major cardiovascular events.^8^

More data is still needed about the SGLT2 inhibitor role in patients with established heart failure, regardless of the presence or absence of T2DM.

### The study

DAPA-HF trial stands for Dapagliflozin in Patients with Heart Failure and Reduced Ejection Fraction. The trial was conducted as a prospective, randomized, placebo-controlled, multicenter trial (in 410 centers in 20 countries). The study was published in *New England Journal of Medicine* in September 2019.^9^

The study enrolled 4,744 patients with symptomatic heart failure and reduced ejection fraction (≤40%), older than 18 years, to assess the efficacy and safety of dapagliflozin in those patients who were already diagnosed to have heart failure - regardless of being diabetic or not.

Patients with hypotension, renal impairment (GFR <30ml/min/1.73 m^2^), or with unacceptable risks of side effects of SGLT2 inhibitors were excluded from the study. 2,373 patients blindly received daily 10 mg of dapagliflozin regardless presence or absence of DM, while 2,371 patients received placebo in addition to standard therapy of heart failure (guideline directed medical therapy, ICD or CRT). The primary endpoint was a composite of worsening of heart failure (hospitalization or an urgent visit resulting in intravenous therapy for heart failure) or cardiovascular-related deaths. The secondary endpoint was worsening of heart failure, CVS death and additionally, total number of hospitalizations with heart failure, change of quality of life using Kansas City Cardiomyopathy Questionnaire (KCCQ), worsening of renal function, renal death or death from any cause. About of 42% of patients in each group had type-2 DM.

**Figure 2.**
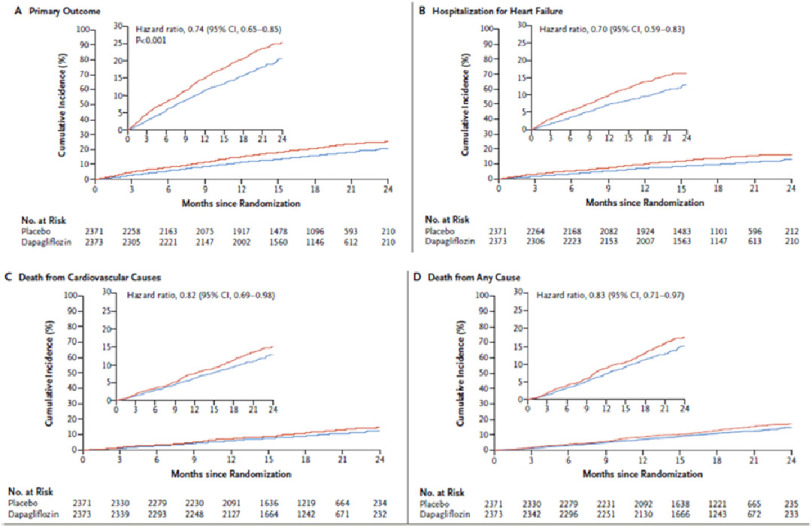
Primary and secondary outcomes in dapagliflozin group versus placebo group.

The results of the trial showed that the dapagliflozin was superior to placebo at preventing cardiovascular deaths and heart failure events. The primary composite outcome of worsening heart failure or death from cardiovascular causes was lower in the dapagliflozin group (16.3%) compared with 21.2% in the placebo group (hazard ratio, 0.74; *p* < 0.001). Of the patients receiving dapagliflozin, 231 (9.7%) were hospitalized for heart failure, as compared with 318 patients (13.4%) receiving placebo. Moreover, death from cardiovascular causes occurred in 227 patients (9.6%) who received dapagliflozin and in 273 (11.5%) who received placebo. All causes of death were lower in the dapagliflozin group than placebo group (11.6% vs. 13.9%) (Figure 2).^10^

Moreover, the number of patients who would need to have been treated with dapagliflozin to prevent one primary event was 21.

The study showed some improvement in patients’ symptoms (using KCCQ) in dapagliflozin group rather than placebo group (58.3% vs. 50.9%; odds ratio, 1.15; 95% CI, 1.08 to 1.23).

The effect of dapagliflozin on the primary outcome was generally consistent across prespecified subgroups, including in patients without diabetes at baseline, although the patients in NYHA functional class III or IV appeared to have less benefit than those in class II.

On the other hand, the study showed that serious renal adverse events occurred in 38 patients (1.6%) in the dapagliflozin group and in 65 patients (2.7%) in the placebo group (*p* = 0.009). Adverse events rarely led to a discontinuation of treatment. All serious adverse events are listed in Table 1. There was no notable excess of any event in the dapagliflozin group.

**Table 1:** Primary and secondary cardiovascular and adverse events in DAPA-HF trial.

**Variable**	**Dapagliflozin (*N* = 2373)**		**Placebo (*N* = 2371)**		**Hazard or Rate Ratio or Difference (95% CI)**	***P* Value**
		events/100 patient-yr		events/100 patient-yr		
**Efficacy outcomes**						
Primary composite outcome—no. (%)	386 (16.3)	11.6	502 (21.2)	15.6	0.74 (0.65 to 0.85)	<0.001
Hospitalization or an urgent visit for heart failure	237 (10.0)	7.1	326 (13.7)	10.1	0.70 (0.59 to 0.83)	NA
Hospitalization for heart failure	231 (9.7)	6.9	318 (13.4)	9.8	0.70 (0.59 to 0.83)	NA
Urgent heart-failure visit	10 (0.4)	0.3	23 (1.0)	0.7	0.43 (0.20 to 0.90)	NA
Cardiovascular death	227 (9.6)	6.5	273 (11.5)	7.9	0.82 (0.69 to 0.98)	NA
Secondary outcomes						
Cardiovascular death or heart-failure hospitalization—no. (%)	382 (16.1)	11.4	495 (20.9)	15.3	0.75 (0.65 to 0.85)	<0.001
Total no. of hospitalizations for heart failure and cardiovascular deaths	567	–	742	–	0.75 (0.65 to 0.88)	<0.001
Change in KCCQ total symptom score at 8 mo	6.1 ± 18.6	–	3.3 ± 19.2	–	1.18 (1.11 to 1.26)	<0.001
Worsening renal function—no. (%)	28 (1.2)	0.8	39 (1.6)	1.2	0.71 (0.44 to 1.16)	NA
Death from any cause—no. (%)	276 (11.6)	7.9	329 (13.9)	9.5	0.83 (0.71 to 0.97)	NA
**Safety outcomes**						
Discontinuation due to adverse event—no./total no. (%)	111/2368 (4.7)	–	116/2368 (4.9)	–	–	0.79
Adverse events of interest—no./total no. (%)						
Volume depletion	178/2368 (7.5)	–	162/2368 (6.8)	–	–	0.4
Renal adverse event	153/2368 (6.5)	–	170/2368 (7.2)	–	–	0.36
Fracture	49/2368 (2.1)	–	50/2368 (2.1)	–	–	1.00
Amputation	13/2368 (0.5)	–	12/2368 (0.5)	–	–	1.00
Major hypoglycemia	4/2368 (0.2)	–	4/2368 (0.2)	–	–	NA
Diabetic ketoacidosis	3/2368 (0.1)	–	0	–	–	NA
Fournier’s gangrene	0	–	1/2368 (<0.1)	–	–	NA
**Laboratory and other measures**						
Change from baseline to 8 mo						
Glycated hemoglobin—%	−1.21 ± 1.14	–	0.04 ± 1.29	–	−0.24 (−0.34 to −0.13)	<0.001
Creatine—mg/dl	0.07 ± 0.24	–	0.04 ± 0.25	–	0.02 (0.01 to 0.03)	<0.007
Hematocrit—%	2.31 ± 3.90	–	−0.19 ± 3.81	–	2.41 (2.21 to 2.62)	<0.001
NT-proBNP—pg/ml	−196 ± 2387	–	101 ± 2944	–	−303 (−457 to −150)	<0.001
Weight—kg	−0.88 ± 3.86	–	0.10 ± 4.09	–	−0.87 (−1.11 to −0.62)	<0.001
Systolic blood pressure—mm Hg	−1.92 ± 14.92	–	−0.38 ± 15.27	–	−1.27 (−2.09 to −0.45)	0.002

The incidence of the pre-specified renal composite outcome did not differ between the treatment groups.

Findings in patients with diabetes were similar to those in patients without diabetes. The frequency of adverse events related to volume depletion, renal dysfunction, and hypoglycemia did not differ between treatment groups.

## Discussion

Sodium–glucose co-transporter-2 (SGLT2) inhibitors canagliflozin, empagliflozin and dapagliflozin, reduced the risk of heart failure hospitalization among patients with type-2 diabetes and cardiovascular disease/cardiovascular risk factors, with an apparently similar treatment effect in the small subgroup (10–15%) of patients in each trial with baseline heart failure of undetermined phenotype, as illustrated in a meta-analysis of EMPA-REG OUTCOME, CANVAS and DECLARE–TIMI 58.^11^

These trials showed a reduction in heart failure hospitalization within weeks to months of randomization. The rapid onset of benefit in the three trials is not consistent with traditional views about the mechanisms and time course of cardiovascular protection accruing with conventional glucose-lowering therapies.^12^

Based on this perspective, numerous additional beneficial mechanisms have been suggested, ranging from diuretic and related hemodynamic actions, effects on myocardial metabolism, ion transporters, fibrosis, adipokines, uric acid, and kidney function.

A recent mediation analysis suggested that the rise in haematocrite concentration following SGLT2 inhibitor treatment is related to benefit, supporting a diuretic action, and mathematical modelling suggests SGLT2 inhibitors may remove fluid preferentially from the interstitial space and cause less intravascular volume contraction.^13^

Other data suggest SGLT2 inhibition can lead to ketogenesis and an increase in hydroxybutyrate, which provides an alternative and more efficient 5substrate for myocardial energy generation.^12^

DAPA-HF is a landmark trial. It took a diabetes drug and used it in patients without diabetes, a concept that would have been considered outlandish five years ago. DAPA-HF results transform dapagliflozin from antidiabetic to heart failure drug.

The conclusion of DAPA-HF Among patients with heart failure and a reduced ejection fraction, the risk of worsening heart failure or death from cardiovascular causes was lower among those who received dapagliflozin than among those who received placebo, regardless of the presence or absence of diabetes. Dapagliflozin may therefore signal a new approach in the treatment of patients with HFrEF.

### What have we learned?

Dapagliflozin reduces morbidity and mortality in symptomatic patients with reduced ejection fraction compared to placebo. It may change the management strategy of heart failure, but the results still need to be reviewed by regulators and guideline writers. However, while SGLT2 inhibitor is a potent, effective drug, its prescription may raise its cost and widespread use could reveal more side effects.
